# Tetralogy of Fallot Will be Treated Interventionally Within Two Decades

**DOI:** 10.1007/s00246-020-02297-z

**Published:** 2020-03-20

**Authors:** Muhammed Riyas K. Rahmath, Younes Boudjemline

**Affiliations:** Sidra Medicine, Heart Center, PO BOX 26999, Doha, Qatar

**Keywords:** Tetralogy of fallot, Transcatheter, Stent, Cardiac catheterization

## Abstract

Tetralogy of Fallot is considered a prototype congenital heart disease because of its embryological, anatomical, pathophysiological, and management aspects. Current management usually relies on a complete surgical repair that is electively performed between 3 and 6 months of age. With the advances of interventional cardiology especially in the fields of ventricular septal defect closure, stent, and pulmonary valve replacement, the question of complete repair of tetralogy of Fallot by interventional means can be discussed. Tetralogy of Fallot is a complex disease with multiple lesions, all individually amenable to transcatheter treatment. In this article, we will review current status of various aspects of tetralogy of Fallot focusing on interventional aspects, giving insights of what would be the ideal platform of a fully interventional repair.

## Introduction

Tetralogy of Fallot is considered a prototype congenital heart disease because of its embryological, anatomical, pathophysiological, and management aspects. Understanding of pathophysiology and management of this disease have evolved over decades. Operated tetralogy of Fallot patients have become a major group of patients in adult congenital heart disease category [1–3]. Clinical spectrum of tetralogy of Fallot varying from severe cyanosis and ductus dependent pulmonary circulation to older patients with subtle cyanosis. Current management usually relies on a complete surgical repair that is electively performed between 3 and 6 months of age. This timing can be overwhelmed in situation where pulmonary blood is not sufficient to provide adequate oxygenation. Care of those patients may be more complicated requiring a multi-disciplinary approach involving cardiologists, interventionists, and cardiac surgeons. Patients with inadequate pulmonary blood flow usually have to go to palliative approach before being amenable to a complete repair. With the advances of interventional cardiology especially in the fields of ventricular septal defect closure, stent, and pulmonary valve replacement, the question of complete repair of tetralogy of Fallot by interventional means can be discussed. Tetralogy of Fallot is a complex disease with multiple lesions, all individually amenable to transcatheter treatment. In this article, we will review current status of various aspects of tetralogy of Fallot focusing on interventional aspects, giving insights of what would be the ideal platform of a fully interventional repair.

## Anatomical Features of Tetralogy of Fallot

**Fig. 1:**
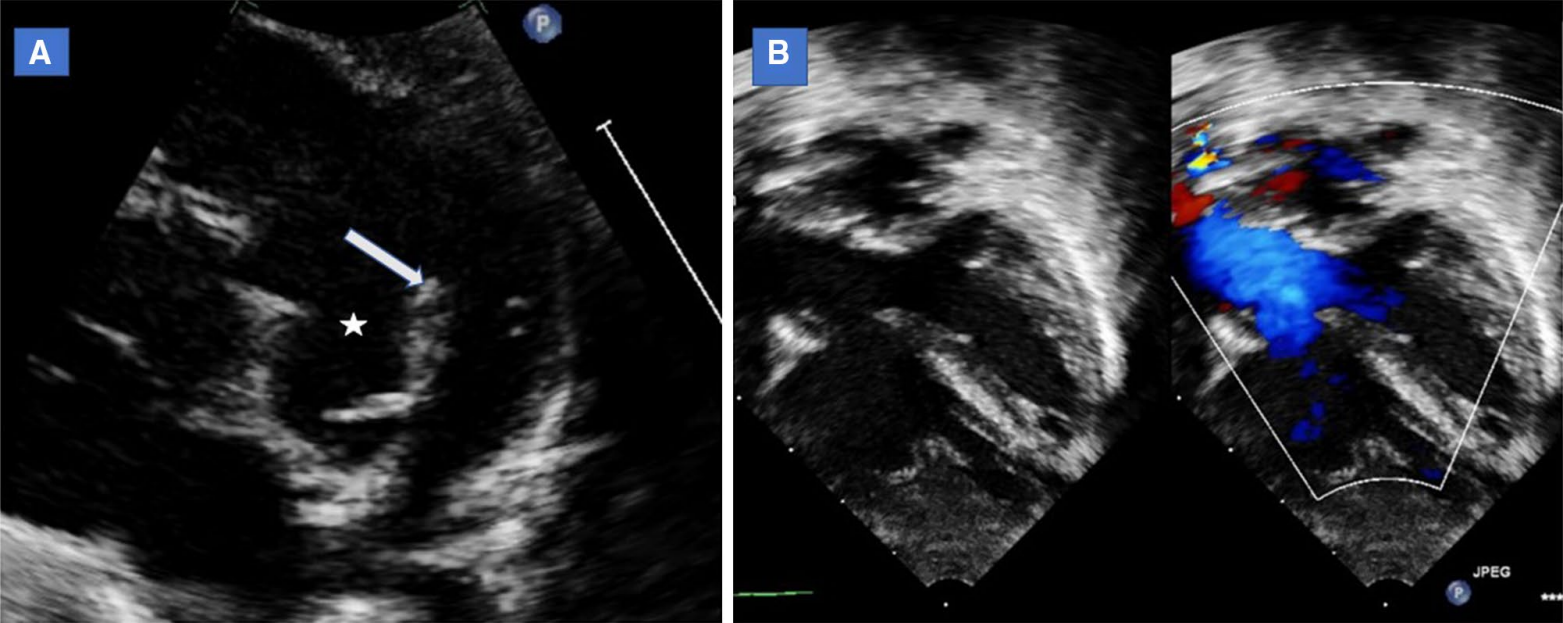
Echocardiographic images showing main features of tetralogy of Fallot. **a** Parasternal short-axis image showing anterior deviation of the conal septum (arrow) and resultant ventricular septal defect shown (asterisk). **b**. Apical five chamber echo view showing aortic override to the right ventricle and maligned ventricular septal defect

Fundamentally, tetralogy of Fallot is a monology as the hallmark lesion is a cephalad and anterior displacement of the infundibular septum resulting in both ventricular septal defect and right ventricular outflow tract obstruction (Fig. 1). However, tetralogy of Fallot represents a wide spectrum of diseases. There might be varying degree of aortic override and condition will be labeled as double outlet right ventricle if there is more than 50% of the aortic valve override to the right ventricle [4]. Obstruction to pulmonary flow in tetralogy of Fallot is usually sub-valvular but other lesions can be present. Pulmonary valve stenosis can rarely be the major cause of obstruction especially in infants. All types of lesions can be seen here from stenosis of a three leaflet valves to hypoplastic annulus with absent leaflets. In addition, stenosis of the pulmonary arteries can also be seen complicating management of those patients.

## Management of Tetralogy of Fallot

Standard treatment of tetralogy of Fallot is surgical correction which consists of ventricular septal defect patch closure and extensive relief of right ventricular outflow tract obstruction. A transannular patch is performed if the pulmonary annulus is too small. Treatment of additional lesions might be needed if present. It is usually carried out in infancy when the age of the patients is 3 to 6 months. Earlier repair has been safely performed without any increase in re-intervention rates irrespective of size of the patient [5–7]. However, most of the centers are still performing complete repair at a later age in asymptomatic patients [8]. Neonatal tetralogy of Fallot repaired patients has higher reoperation rate, higher incidence of transannular patch, and higher mortality [9–11]. This explains why most centers prefer a palliative approach as initial therapy before offering complete repair in younger age group. Palliative procedure is either surgical or interventional. Possible interventions will be discussed in the next paragraph. Palliative procedures are aimed to improve oxygen saturations, so that the definitive procedures can be performed at a later stage with lesser risk. Surgical options are right ventricular outflow tract opening or Blalock–Taussig shunt [12, 13]. These surgeries improve pulmonary blood flow and promote growth of the pulmonary arteries allowing complete repair at a later stage in most cases.

## Role of Cardiac Catheterization in Patients with Tetralogy of Fallot

**Fig. 2:**
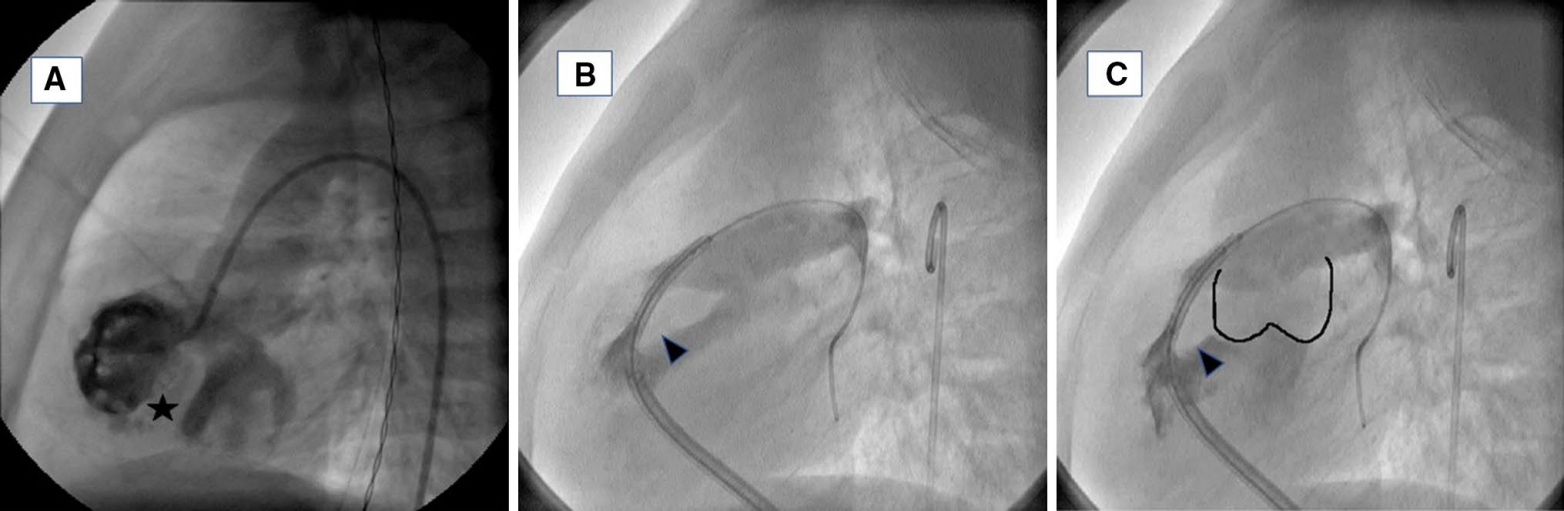
Angiographic images showing main features of tetralogy of Fallot. **a** Right ventricular injection showing the inter-ventricular septum (asterisk) and the overriding of the aorta. **b** Right ventricular outflow tract angiogram showing the anteriorly deviated conal septum (arrow head) causing significant narrowing of the right ventricular outflow tract. **c** Right ventricular outflow tract angiogram showing anteriorly deviated conal septum and aortic sinuses marked in black line

**Fig. 3:**
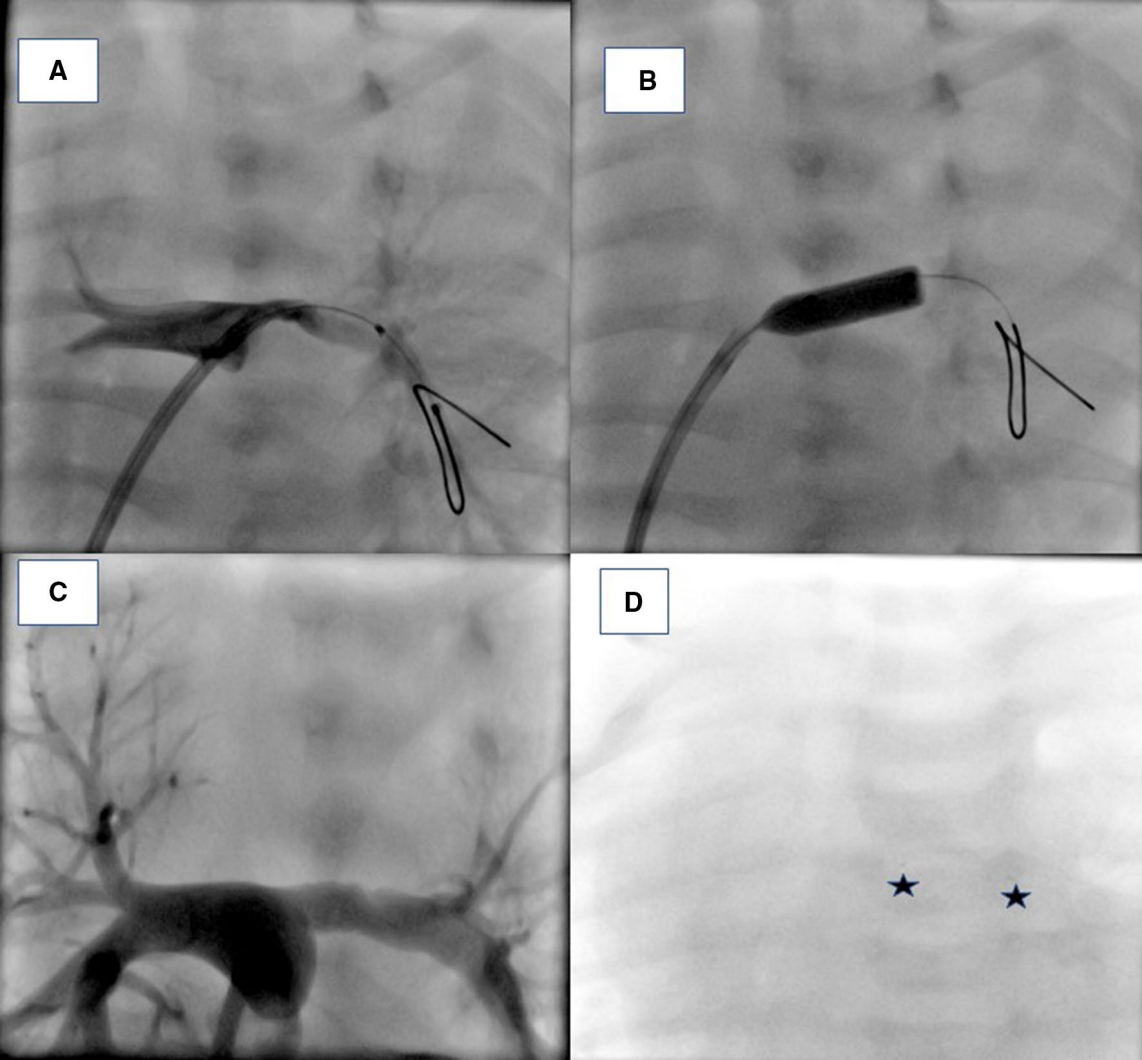
Angiographic images in a patient following surgical repair of a tetralogy of Fallot. **a** Left pulmonary artery angiogram showing stenosis at the origin of the left pulmonary artery. **b** Placement of biodegradable stent (Abbott, Absorb). **c** Angiogram following stent placement showing complete opening of the stenosis. **d** Fluoro image showing radio-opaque dots (shown by two asterisk) to identify the position of the biodegradable stent in the left pulmonary artery

Over the last decade and with the advance of non-invasive imaging (i.e., echocardiography, computed tomography angiography or magnetic resonance imaging), the role of cardiac catheterization in most congenital heart diseases and in tetralogy of Fallot in particular has shifted from diagnostic to interventional. Nowadays, a diagnostic catheterization is seldom indicated in tetralogy of Fallot patients (Fig. 2) [14]. A large spectrum of interventions can be performed in tetralogy of Fallot patients. After palliative repair, patients who have an occlusive thrombus in Blalock–Taussig shunt can undergo an emergency relief of obstruction usually with stent implantation. After the complete surgical repair, cardiac catheterization can be done to treat residual lesions or long-term complications such as pulmonary artery stenosis (balloon angioplasty or stent implantation), residual or additional ventricular septal defect (closure using devices), or pulmonary valve regurgitation (percutaneous pulmonary valve implantation) (Fig. 3).

**Fig. 4:**
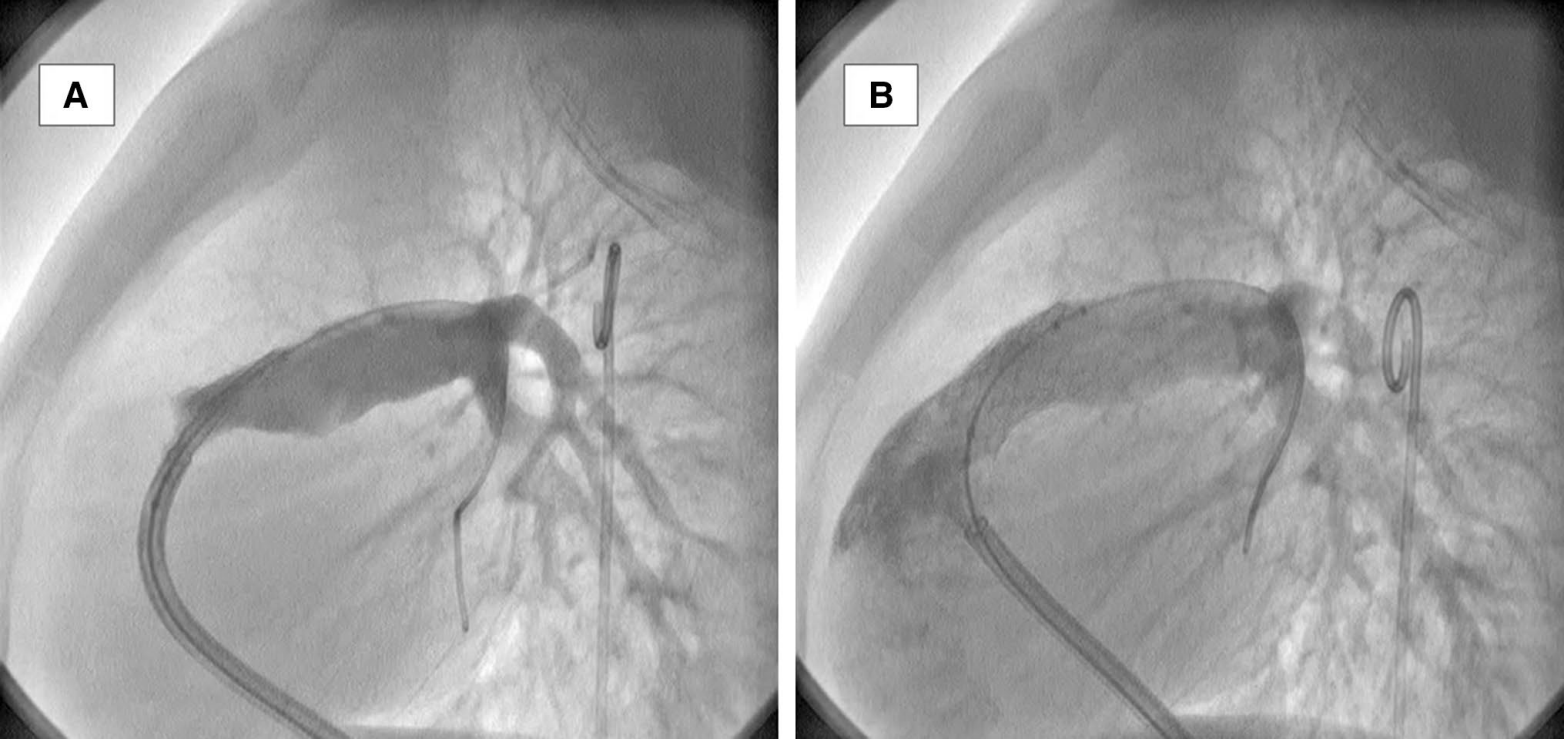
Angiographic images in a patient with a tetralogy of Fallot. **a** Main pulmonary angiogram showing narrow outflow tract and pulmonary annulus, hypoplastic main and branch pulmonary arteries. **b** Angiogram following placement of stent in the right ventricular outflow tract extending to pulmonary annulus

In native tetralogy of Fallot, cardiac interventions can also be performed mimicking what is done in surgery but no transcatheter complete repair has been reported so far. Palliative approaches aiming to improve oxygenation and pulmonary blood flow are performed on regular basis. Right ventricular outflow tract stenting (Fig. 4) or pulmonary valve balloon dilatation (as an alternative to surgical right ventricular outflow tract opening), and transcatheter placement of a stent in a patent arterial duct (as an alternative to systemic shunt) can be offered to symptomatic patients [15, 16]. The choice between techniques depends on multiple factors including operator’s or center preferences, stent availability, age, patency of the ductus, and location/extent of the stenosis. If the right ventricular outflow tract obstruction is predominantly due to valvar stenosis, percutaneous ballooning of the pulmonary valve can alleviate the stenosis and postpone the need for surgery and interestingly, during complete surgical repair, some surgeons use this approach to reduce the need for transannular patch (valve sparing) [17]. However, when the right ventricular outflow tract obstruction is at the infundibular level, balloon dilatation will not provide good relief of obstruction and often placement of a stent is required [12, 13]. This approach can also be used as a good palliative measure in adults who is not willing to have complete repair. The aim of this procedure is to increase the pulmonary blood flow but leaving some stenosis to avoid systemic pressure to be transmitted to the pulmonary bed. Diameter of the stent will depend on the age and weight of the patient but usually range from 4  (in neonates) to 14 mm (in adults). After infancy when diameter of the stent is uncertain, the practice is to start at low diameter and post-dilate the stent until appropriate/desired saturation is obtained keeping the pulmonary artery pressures normal. However, when stent implanted in infancy, it has been shown that it increases the need for transannular patch that has deleterious effects in the long term. For this reason, in our opinion, patent ductus arteriosus stenting should be preferred every time the pulmonary blood flow relies on the patent ductus arteriosus. Patent ductus arteriosus stenting is safer to perform in tetralogy of Fallot patients (as there is forward flow across the right ventricular outflow tract) unlike in babies with pulmonary atresia (in which patent ductus arteriosus is the only source of pulmonary flow which can result in significant desaturation during the procedure). An arterio-venous circuit is realized allowing for precise and safe placement of stent. Like with any patent ductus arteriosus stent, this approach promotes and allows symmetrical growth of both pulmonary arteries.

## Hurdles to be Considered When Thinking About Complete Repair of Tetralogy of Fallot by Transcatheter Technique

As mentioned previously, there is no report of a complete transcatheter tetralogy of Fallot repair so far. Anatomical features should be discussed further to understand present limitations. The hallmark lesion of tetralogy of Fallot is a superior and anterior displacement of the infundibular septum. Therapies are focused on treating features including both ventricular septal defect and right ventricular outflow tract obstruction. Theoretically both anomalies are can be treated by interventions. Device closure of muscular ventricular septal defects and selected cases of peri-membranous ventricular septal defects have already been established as a mode of treatment. However, closure of ventricular septal defect in tetralogy of Fallot patients with device has technical difficulties. Being a malaligned ventricular septal defect, mostly it is large in size and has deficient aortic rim. Associated overriding of aorta (as in extreme cases of double outlet right ventricle) also imposes difficulty to consider device closure of such ventricular septal defects using the conventional approach. Though it’s not common, ventricular septal defect in tetralogy of Fallot can be doubly committed which again make the closure of ventricular septal defect difficult by commercially available devices. In addition, the right ventricular outflow tract obstruction in tetralogy of Fallot patients is complex in nature being in most cases a combination of sub-valvar, valvar, and supra-valvar. Supra-valvar stenosis can be taken care to a good extent by ballooning or stent placement. Transcatheter infundibular right ventricular outflow tract obstruction management (mainly by stent implantation) might give satisfactory results in the short-term follow-up. However, because it carries risk of stent fracture, sub-valvar obstruction involving muscle hypertrophy do not give good outcome in the long term. For this reason, any sub-valvular stenosis is usually considered as a surgical anomaly.

If technically imaginable, these approaches will have to show feasibility and safety comparable to current standard of care. Reaching surgical results will be difficult as mortality and reoperation rate is quite low in tetralogy of Fallot repair in standard situations. The tendency in this situation has always been to start such procedure in sicker patients. For tetralogy of Fallot patients, this will increase the complexity of any complete transcatheter approach as symptomatic patients are usually younger with low weight and both these features are limiting factors in any transcatheter procedures. In tetralogy of Fallot repair, it is not only a problem with vascular access, also because any device placed at lower age has to follow the growth or be present until the anomalies are reversed. By starting the interventional procedures with the population of older patients living on an unrepaired tetralogy of Fallot (palliated or not) might be a solution to test the feasibility of the procedures and ideas.

## Future Approaches to Treat Tetralogy of Fallot by Interventional Means

### Imaging and Helping Tools

The utilization of 3D printing in interventional Cardiology is expending especially in the field of transcatheter pulmonary valve replacement. Use of 3D technology (including printing) has been shown for describing the vessels anatomy and guiding the surgical approach for intra-cardiac complex anatomy [18, 19]. The 3D anatomical models showed a trend for reduced operating room time when compared with similar surgeries. This could have significant impact on patient outcomes and operating room economics [20]. 3D printed models can also be used for rehearsal of the planned procedure before the actual surgery so that the procedure can be optimally tailored. It helps for understanding of complex anatomical spatial relationships. 3D models provided better anatomic delineation and additional information that altered or confirmed the surgical plan [21]. 3D printed models enhance resident education on tetralogy of Fallot by improving learner satisfaction [21]. In tetralogy of Fallot patients, 3D models might help to develop a fully transcatheter approach for complete repair and test possible devices and approaches. Animal models of tetralogy of Fallot are also available.

### Possible Approaches for Complete Repair

Considering a full transcatheter repair, tetralogy of Fallot should also be seen as a monology. Being able to use a single device, which can realign the anteriorly displaced septum that causes right ventricular outflow tract obstruction, might be a solution. By shifting the septum, the right ventricular outflow tract obstruction would be relieved. Displacing the septum to its correct position would probably not be enough to close the ventricular septal defect at the same time but would offer an adequate rim for the closure of the residual ventricular septal defect using ventricular septal defect occluders. A single (covered) device with the shape of a bottleneck or opened with a balloon to obtain such a shape would be able to treat all tetralogy of Fallot anomalies at the same time if long enough to cover the whole ventricular septal defect. Otherwise, ventricular extension of such device would act as a rim for any occluder. Relieving the right ventricular outflow tract obstruction and decreasing the right ventricular systolic pressure (by complete or near-complete closure of the ventricular septal defect) would allow the regression of the right ventricular hypertrophy. However, because standing in the right ventricular outflow tract, that kind of device would be under very high stress, prone to fracture and might be the substrate for arrhythmias. Of course, like any procedure resulting in expansion of the right ventricular outflow tract, anatomy of the coronaries should be looked at to make sure that no compression will occur after complete right ventricular outflow tract obstruction relief or device expansion. Another possible way (non-mechanic) of dealing the right ventricular outflow tract obstruction would be to act directly on the septal muscle by initiating and creating necrosis similarly to what is done for hypertrophic cardiomyopathy with left ventricular outflow tract obstruction. Alcohol injection or coil occlusion of the coronary septal branch feeding the obstructing septum or septal ablation (using cryo or laser ablation or any other technique) is some of the possibilities to get rid of right ventricular outflow tract obstruction. This would leave the patient with a maligned ventricular septal defect and possible annular and supra-valvular pulmonary stenosis. A single device in form of a stent hanging in the annulus or supra-annular position with extension to close the ventricular septal defect would take care of the remaining anomalies. Because the device would be mechanically fixed in the main pulmonary artery, a single disk technology with or without hooks placed in the left ventricle would avoid risk of device fracture in the sub-valvular area. Type of material to use for such a device is questionable. Of course, a fully bioresorbable device would be theoretically very attractive seen as the grail. However, results with bioabsorbable devices have been disappointing so far. In this situation, high radial strength for stent part is needed. Poly(l-lactic) acid does not provide such mechanical characteristics. Metallic bioresorbable stents might be more appropriate but this technology is just emerging and not available in clinical practice. Finally, having a non-resorbable stent in the pulmonary artery fixing the annulus might also be interesting in the long term to avoid inappropriate dilatation of the main pulmonary artery and provide a good leading zone for a transcatheter pulmonary valve.

## Conclusion

Surgical repair of tetralogy of Fallot provides excellent results in the current era. With the recent advances in interventional cardiology in simple cardiac anomalies, the question of complete repair of more complex anomalies like tetralogy of Fallot has been raised. We reviewed current status of various aspects of tetralogy of Fallot focusing on interventional aspects, giving insights of what would be the ideal platform of a fully interventional repair. With the help of 3D modeling and the development of new materials such as bioresorbable devices, we are convinced that, in coming two decades, many advances will be made to interventionally approach more complex congenital heart disease like the tetralogy of Fallot.
